# Short ELF-EMF Exposure Targets SIRT1/Nrf2/HO-1 Signaling in THP-1 Cells

**DOI:** 10.3390/ijms21197284

**Published:** 2020-10-02

**Authors:** Patruno Antonia, Costantini Erica, Ferrone Alessio, Pesce Mirko, Diomede Francesca, Trubiani Oriana, Reale Marcella

**Affiliations:** 1 Department of Medicine and Science of Aging, University “G. D’Annunzio”, 66100 Chieti, Italy; antonia.patruno@unich.it (P.A.); alessioferrone@yahoo.it (F.A.); mirko.pesce@unich.it (P.M.); 2 Department of Medical, Oral and Biotechnological Sciences, University “G. D’Annunzio”, 66100 Chieti, Italy; francesca.diomede@unich.it (D.F.); oriana.trubiani@unich.it (T.O.); Marcella.reale@unich.it (R.M.)

**Keywords:** extremely low frequency electromagnetic fields (ELF-EMF), heme oxygenase-1 (HO-1), nuclear factor (erythroid-derived 2)-like-2 factor (Nrf2), silent information regulator 1 (SIRT1), THP-1 cells

## Abstract

Extremely low frequency electromagnetic fields (ELF-EMFs) have been known to modulate inflammatory responses by targeting signal transduction pathways and influencing cellular redox balance through the generation of oxidants and antioxidants. Here, we studied the molecular mechanism underlying the anti-oxidative effect of ELF-EMF in THP-1 cells, particularly with respect to antioxidant enzymes, such as heme oxygenase-1 (HO-1), regulated transcriptionally through nuclear factor E2-related factor 2 (Nrf2) activation. Cells treated with lipopolysaccharides (LPS) were exposed to a 50 Hz, 1 mT extremely low frequency electromagnetic fields for 1 h, 6 h and, 24 h. Our results indicate that ELF-EMF induced HO-1 mRNA and protein expression in LPS-treated THP-1 cells, with peak expression at 6 h, accompanied with a concomitant migration to the nucleus of a truncated HO-1 protein form. The immunostaining analysis further verified a nuclear enrichment of HO-1. Moreover, ELF-EMF inhibited the protein expressions of the sirtuin1 (SIRT1) and nuclear factor kappa B (NF-kB) pathways, confirming their anti-inflammatory/antioxidative role. Pretreatment with LY294002 (Akt inhibitor) and PD980559 (ERK inhibitor) inhibited LPS-induced Nrf2 nuclear translocation and HO-1 protein expression in ELF-EMF-exposed cells. Taken together, our results suggest that short ELF-EMF exposure exerts a protective role in THP-1 cells treated with an inflammatory/oxidative insult such as LPS, via the regulation of Nrf-2/HO-1 and SIRT1 /NF-kB pathways associated with intracellular glutathione (GSH) accumulation.

## 1. Introduction

The immune response is a signal-mediated reaction to tissue invasion by pathogens, toxins, physical injury, or stress. It is triggered by signal transduction, which mediates changes to cell metabolism, and gene transcription, which leads cell response to environmental stimuli. Cellular signaling is part of a complex communication system that regulates basic cellular activities and coordinates cellular actions. The activation of the immune system is mediated by “warning signs” that are important for the initiation of immune-inflammatory and anti-inflammatory responses necessary for recovery of homeostasis.

Inflammation plays a significant role against infections, in response to injury, and also in the onset and progression of degenerative diseases, and macrophages are key players in the regulation of innate and specific immune response, immune surveillance, wound healing, and the maintenance of homeostasis. The dysregulation of macrophage activation states is linked to inflammatory diseases, autoimmunity, cancer, and chronic wounds.

In the progression of many inflammatory diseases, reactive oxygen species (ROS) may play a key role, and monocytes respond to oxidative stress with an upregulation of enzymes involved in adaptive survival response. A set of genes coding for enzymes induced by oxidative stress, called phase 2 proteins, share a protective function against electrophiles and ROS, although their protection mechanisms are different. Heme oxygenase-1 (HO-1) (~32 kda, enzyme classification (EC) 1.14.99.3), encoded by the HMOX1 gene, is one of the phase 2 enzymes, and it has been proposed as a marker of oxidative stress because it is readily induced in several cell types by its substrate heme and by a range of stress stimuli [1,2].

Recently, the opportunity to use electromagnetic fields (EMFs) exposure to modulate immune cell responses has been suggested and discussed with increasing interest [3,4].

The effects of exposure to extremely low frequency (ELF)-EMFs on human health have been assessed for a long time with in vivo, in vitro, and epidemiological studies, focusing on the possible relationship between ELF-EMF and a variety of biological processes. Studies have yielded different results that often conflict with each other, and no consensus has been reached [5,6]. The biological effects of exposure to ELF-EMFs have been investigated in many biological targets related to the immune system, and it has been understood that harmful or therapeutic effects depend on the frequency, amplitude, field strength, and duration of exposure, as well as on the characteristics of the targeted cell types.

In in vitro experiments carried out in our laboratory, we found that HaCaT cells exposed to 50 Hz, 1 mT flux density, already upregulate matrix metalloprotease (MMP) 9 level at 1 h, peaking at 8 h, and decreasing at 24 h, in accordance to the wound healing [7]. The increase of the canonical pathway of the mammalian target of rapamycin (mTOR) regulation phosphoinositide 3-kinases/protein kinase B (PI3K/Akt) and activation of extracellular signal-regulated kinases (Erk) signaling pathways were detected in HaCaT cells exposed to 50 Hz, 1 mT, for 1–3 h [8]. In SH-SY5Y cells, short ELF-EMF exposure increased the intracellular 5-hydroxyindoleacetic acid/5-hydroxytryptamine ratio [9,10]. Also, a rapid and sustained elevation in nitric oxide synthetase (NOS) activity, a time-dependent elevation in catalase (CAT) activity, and increased cytochrome (CYP) 450 activity and O_2_^−^ production were observed [11]. A significant modulation of iNOS, CAT, and CYP 450 protein expression was recorded in cells exposed to ELF-EMF [11,12].

In the present study, we investigated the effect and the action mechanism of 50 Hz ELF-EMF on monocytic leukemia THP-1 cells, which are accepted as a good model of human monocytes/macrophages [13].

For a better understanding and description of the potential effects of exposure, lipopolysaccharides (LPS) treatment was used as a positive control of monocyte activation. We first examined the effect of the exposure on SIRT1 /Nrf2/HO-1 expression and oxidative balance on THP-1 cells. Next, interference of the inhibitor of Erk and Akt was used to study the likely mechanism of ELF-EMF on the oxidative balance in THP-1 cells.

## 2. Results

### 2.1. Effects of ELF-EMF on HO-1 Expression and Nuclear Localization

**Figure 1:**
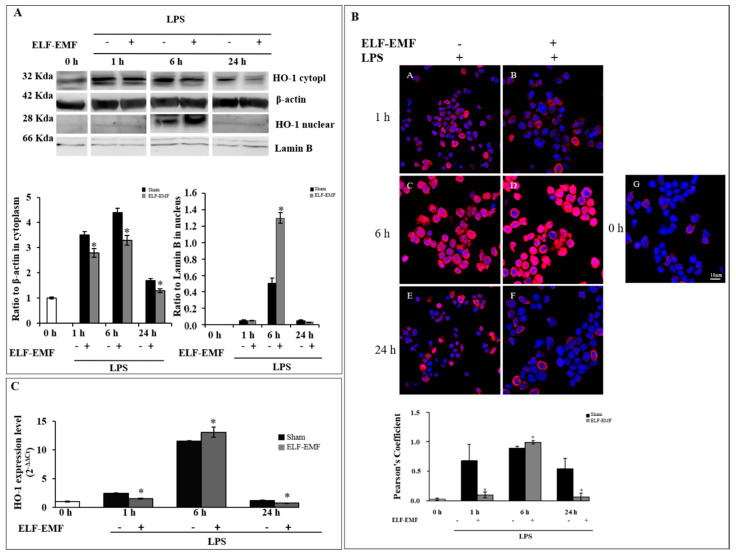
Extremely low frequency electromagnetic field (ELF-EMF) effects on nuclear translocation of heme oxygenase-1 (HO-1) in THP-1 cell line. (**A**) Representative image of western blot analysis of HO-1 protein in cytoplasmic and nuclear fractions extracted from THP-1 cells cultured with lipopolysaccharides (LPS) and ELF-EMF-exposed or sham-exposed (top). Averaged band density of HO-1 normalized vs. β-actin for cytoplasm and vs. Lamin B for nucleus (bottom). Data are expressed as mean ± *SD* of three independent experiments performed in triplicate. * *p* < 0.05 ELF-EMF-exposed vs. sham-exposed cells. (**B**) Fluorescence expression at nuclear level and co-localization analyses. Immunolabeling with TO-PRO (blue fluorescence) and heme oxygenase-1 (red fluorescence) in THP-1 cells at basal condition at T0 (g), in LPS-treated cells exposed to ELF-EMF at 1 h (b), 6 h (d), and 24 h (f) or sham-exposed (a,c,e). Bars represent HO-1 co-expression reported as Pearson’s correlation coefficient. * *p* < 0.05 ELF-EMF-exposed vs. sham-exposed cells. The Supplementary materials illustrates the single fluorescence channels used. (**C**) The mRNA expression of HO-1 in THP-1 cells exposed to ELF-EMF and compared to sham-exposed cells. HO-1 expression levels (2^−∆∆*C*t^) are reported as mean ± standard error (*SE*). * *p* < 0.05 ELF-EMF vs. sham assumed as 1.

HO-1 protein and mRNA expression were evaluated in lipopolysaccharides (LPS)-treated THP-1 cells that were either ELF-EMF-exposed or sham-exposed for different amounts of times (1 h, 6 h, and 24 h) (Figure 1).

In Figure 1A, we showed cytoplasmic and nuclear HO-1 protein levels analyzed by western blot in LPS-treated ELF-EMF-exposed or sham-exposed THP-1 cells. Exposure to 50 Hz, 1 mT, for 1 h and 6 h significantly reduced cytoplasmatic HO-1 protein levels in LPS-treated THP-1 cells compared to sham-exposed cells. Interestingly, in the cytoplasm after 1 h of exposure, we detected the presence of 32 kda isoform with an additional faster migrating band at 28 kda. Interestingly, after 6 h, we observed increased nuclear 28 kda HO-1 isoform protein mainly in ELF-EMF-exposed cells. In both exposed and sham-exposed cells, a drastic reduction of cytoplasmatic and nuclear protein was observed after 24 h. After immunolabeling with TO-PRO and heme oxygenase-1 in LPS-treated and exposed or sham-exposed cells, a significantly increased intensity of HO-1 nuclear staining was detected in LPS-stimulated cells after 6 h of exposure (Figure 1B). In Figure 1C, we reported the HO-1 mRNA levels in exposed or sham-exposed and LPS-treated THP-1 cells evaluated with real-time PCR. A significant increase of HO-1 mRNA expression was observed after 6 h of exposure, as well as an increase in the nuclear 28-kDa HO-1 protein level.

### 2.2. Effects of ELF-EMF on Nrf-2 and SIRT1 /NF-kB Pathway

**Figure 2:**
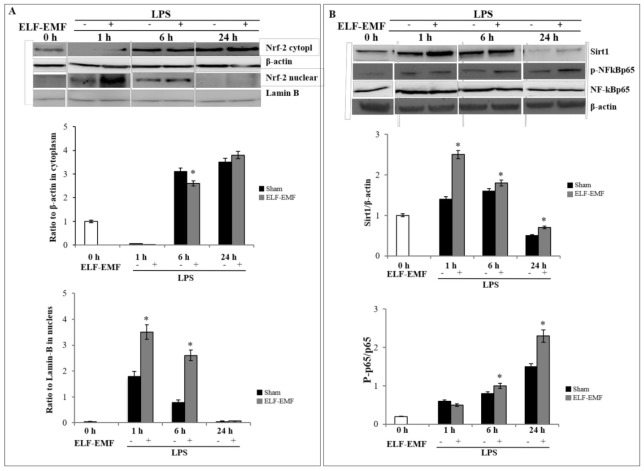
ELF-EMF effects on nuclear factor erythroid 2-related factor 2 (Nrf2) and sirtuin 1 (SIRT1)/ nuclear factor kappa B (NF-kB) pathway.(**A**) Representative western blots (top) and data analysis (bottom) of Nrf2 analyzed in cytoplasmic and nuclear extracts from THP-1 cells treated with LPS and ELF-EMF-exposed or sham-exposed. β-actin and lamin B were used as the internal standards for cytoplasmatic and nuclear fraction, respectively. (**B**) Representative western blots (top) and densitometric analysis (bottom) of Sirt-1, cytoplasmatic NF-kBp65, and nuclear phosphorylated NF-kBp65 protein level in THP-1 cells treated with LPS and ELF-EMF-exposed or sham-exposed. Data are normalized vs. β-actin for Sirt-1 and to respective unphosphorylated protein for p-65 subunit. Data are expressed as mean ± *SD* of three independent experiments performed in triplicate. * *p* < 0.05 ELF-EMF-exposed vs. sham-exposed cells.

Since the cytoprotective protein HO-1 was regulated by nuclear factor erythroid 2-related factor 2 (Nrf2), a redox-sensitive transcription factor, we investigated the Nrf2 protein expression in exposed and sham-exposed THP-1 cultures after different exposure durations (1 h, 6 h, and 24 h). Data reported in Figure 2A show a time-dependent Nrf2 protein nuclear translocation, with a peak following 1 h of exposure and a progressive decline at 6 h and 24 h of exposure.

When we investigated the time-dependent effects of exposure on NAD-dependent deacetylase sirtuin-1 (SIRT1)/nuclear factor kappa B (NF-kB) pathway activation, decreased SIRT1 protein levels and a simultaneous and significant increase of the nuclear translocation of p-NF-kB p65 were observed. In unstimulated cells, NF-κB subunits were located in the cytoplasm. Stressful stimuli induce NF-κB signaling by phosphorylation of the p65 subunit, which is essential for its cytoplasmic to nuclear localization and initiation of the transcription of downstream target genes. Thus, we have assessed the phosphorylation of p65 and its cellular localization by monitoring the levels of p-p65 protein in nuclear extracts by western blot analysis.

### 2.3. ELF-EMF Activates Nrf2/HO-1 Signaling via Akt and ERK 1/2 Pathways

To clarify the upstream signaling pathway involved in the ELF-EMF induction of HO-1 protein and Nrf-2-mediated pathways, we examined ERK and Akt activation, with ERK and Akt regarded as signaling molecules that are mainly involved in HO-1 expression and in cellular protection against oxidative stress.

**Figure 3:**
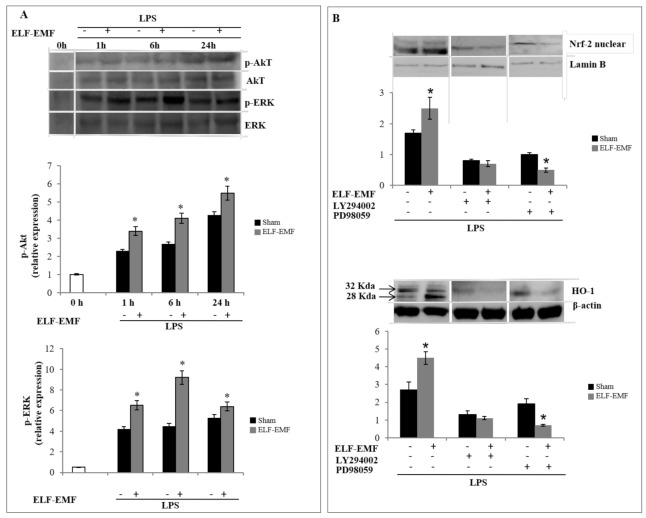
Effect of ELF-EMF exposure on Akt and ERK pathways. (**A**) Analysis of the phosphorylation levels of Akt and ERK in THP-1 cells treated with LPS and ELF-EMF-exposed or sham-exposed by western blotting. (**B**) Representative image of immunoblotting for Nrf-2 and HO-1 in LPS-treated and ELF-EMF-exposed or sham-exposed THP-1 cells for 6 h, pre-treated or not with selective inhibitors of Akt (Ly294002, 1 μmol/L) and ERK (PD980559, 1 μmol/L). Data are reported as the relative expression of nuclear Nrf-2 vs. lamin B and whole cell lysate HO-1 vs. β-actin. Data are expressed as mean ± *SD* of three independent experiments performed in triplicate. * *p* < 0.05 ELF-EMF-exposed vs. sham-exposed cells.

Firstly, we assessed the activation of Akt and Erk proteins by western blot using phospho-specific antibodies against both enzymes at different exposure times (1 h, 6 h, and 24 h). As shown in the densitometric analysis reported in Figure 3A, ELF-EMF exposure caused an increase in the phosphorylation of Akt and Erk compared to LPS-treated cells.

A time-dependent expression of Akt phosphorylation was observed in LPS-treated and ELF-EMF-exposed cells. No significant differences were detected following 1 h and 6 h of exposure, while higher levels of phosphorylation were recorded at 24 h of exposure compared to sham-exposed cells.

In LPS-treated and ELF-EMF-exposed cells, we observed a significant increase in ERK activation/phosphorylation at 1 h, with the peak at 6 h.

After 6 h of ELF-EMF exposure, we observed that HO-1 expression was higher and associated with a nuclear translocation. Thus, we analyzed the potential involvement of AKT and ERK signaling pathways in the ELF-EMF-mediated HO-1/Nrf2 expression in THP-1 cells by using the kinase-specific inhibitors LY294002, (Akt inhibitor) and PD980559 (ERK inhibitor).

Our results also showed that LY294002 and PD98059 strongly inhibited LPS-induced Nrf2 nuclear translocation and HO-1 protein expression in ELF-EMF-exposed cells (Figure 3B), and the major inhibitory effect was obtained with PD980559, an ERK-specific inhibitor, in ELF-EMF-exposed and LPS-treated cells, confirming the effects of ELF-EMF on AKT and ERK signaling pathways.

### 2.4. Effects of ELF-EMF Redox Status and GSH-Related Enzymatic Activities

**Figure 4:**
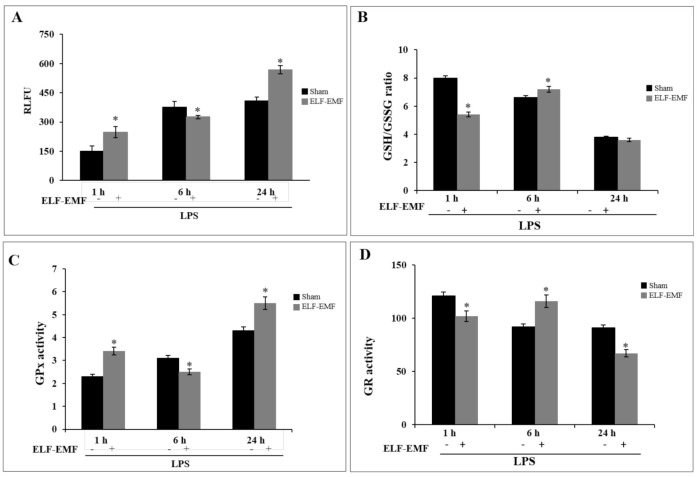
Effect of ELF-EMF exposure on intracellular reactive oxygen species (ROS) formation and glutathione-redox status. (**A**) Intracellular ROS was quantified by 2′,7′-dichlorodihydrofluorescein diacetate (H_2_DCF-DA) probe measuring fluorescence intensity. Data are expressed as the fold increase of ROS production in ELF-EMF-exposed cells relative to the sham-exposed ones. (**B**) Cells were lysed and the intracellular concentration of glutathione (GSH)/oxidized glutathione (GSSG) in all groups was quantified following enzymatic recycling assay. (**C**) Glutathione peroxidase and (**D**) Glutathione reductase activity was normalized with respect to the total protein content of cell lysates. One unit of enzyme activity was defined as 1 µmol of NADPH oxidized/min. Data are expressed as mean ± SD of three independent experiments performed in triplicate. * *p* < 0.05 ELF-EMF-exposed vs. sham-exposed cells. GPx: glutathione peroxidase; GR: glutathione reductase.

As reported in Figure 4A, level analysis of intracellular ROS by DCFH-DA flow cytometric assay showed increased ROS generation in LPS-treated THP-1 cells exposed to ELF-EMF at 1 h, with the peak at 24 h of exposure, compared to sham-exposed cells.

In addition, we examined the effect of ELF-EMF exposure on intracellular glutathione-redox balance, expressed as the ratio of intracellular reduced glutathione (GSH) and oxidized glutathione (GSSG), which plays a critical role in regulating the immune response.

Upon ELF-EMF exposure and LPS treatment, THP-1 cells showed early GSH depletion at 1 h, confirming the oxidant state of the GSH/GSSG balance. At 6 h of exposure, the GSH/GSSG balance showed a reducing state due to a restoration of intracellular GSH levels. However, an increase in the intracellular GSSG level shifted the redox balance toward a significantly oxidizing state at 24 h, suggesting an oxidative cellular state (Figure 4B). Intracellular antioxidant enzyme activities involved in the GSH–redox homeostasis (glutathione peroxidase (GPx) and glutathione reductase (GR)) were evaluated. In LPS-treated THP-1 cells, an increased time-dependent GPx activity was detected, and exposure to ELF-EMF modulated such activity, with an increase at 1 h, a decrease at 6 h, and an increase at 24 h of exposure with respect to sham-exposed cells (Figure 4C). An inverse trend was observed for GR activity in both LPS-treated and exposed cells (Figure 4D).

## 3. Discussion

The effects of ELF-EMFs on biological systems such as immune cells have received extensive interest. Other than the possible negative health impacts, the possibility of ELF-EMFs to favorably modulate immune responses has also been evaluated [14]. Studies have been consistent in stating that the effects of ELF-EMFs vary with frequency, amplitude, and duration of exposure, as well as with the characteristics of the cell types. However, alarms on the potential harmful effects of ELF-EMF exposure on human health were elevated, despite contradictory results preventing the reaching of a conclusive consensus. On the other hand, recent results of many studies have led some researchers to propose and debate the possibility of using ELF-EMF exposure to modulate immune cell responses [4].

The immune system employs many potent effector mechanisms against different types of infections or dangerous stimuli. It is well known that molecules released by infected or injured and damaged cells are responsible for signals that can activate an immune response and subsequent homeostatic mechanisms that drive the pro- or anti-inflammatory balance.

Monocytes and macrophages are important players in the orchestration of inflammatory reactions, and a large number of studies have demonstrated that in response to LPS, monocytes and macrophages produce pro-inflammatory mediators, but also regulatory proteins that counteract the inflammation and oxidative stress.

Due to their uniform genetic background, THP-1 cells are one of most widely used cell lines to investigate the function and regulation of monocytes and macrophages and the analysis of gene expression, protein, and transcription factors of THP-1 cells shed new light on the regulatory mechanism of the monocytes and macrophages in response to inflammatory mediators [15]

Thus, in this study, we have highlighted that in THP-1 cells treated with an inflammatory/oxidative insult, such as LPS, a short ELF-EMF exposure mediated Nrf2 activation and subsequent HO-1 induction, exerting a protective role, partly due to intracellular GSH accumulation.

The activating ability of ELF-EMF on THP-1 cells was shown in our previous study, where redox-related cellular changes involving free radical production with a specific activation of NADH-oxidase were observed [16].

These results are in line with a study by Lupke and co-workers that described human monocytes activation by ELF via a receptor-dependent pathway, similar to bacterial LPS, showing an ELF-induced ROS release of the same magnitude as LPS [17].

ELF-EMF may be able to restore the equilibrium between free radicals and antioxidants, controlling the cascade of inflammatory progression. In fact, several studies have showed that the exposure of different cell types to a 50-Hz ELF-EMF induced an increase in intracellular ROS levels [11,18].

Rollwitz et al. showed that exposure to 50 Hz, 1 mT, stimulated, in mouse macrophages, the NADH oxidase pathway to produce superoxide anion radicals, but not the nicotinamide adenine dinucleotide phosphate oxidase (NADPH) pathway [19]. Frahm et al. reported that, in mouse macrophages, the same exposure conditions increased ROS, HSP70, and HSP110 levels [20]. On the other hand, other studies have not found such an increase, or detected reduced ROS levels in several cell lines exposed to low-intensity ELF-EMFs [21,22].

Heme oxygenase-1 is a Nrf2-regulated gene, responsible for antioxidant, anti-inflammatory, antiapoptotic, antiproliferative and immunomodulatory effects, that is over-activated in the presence of cellular stresses such as inflammation, ischemia, hypoxia, hyperoxia, hyperthermia, or radiation. HO-1 antioxidant and anti-inflammatory properties may be crucial in the development and treatment of several diseases, and the immunomodulatory effects of HO-1 have been shown in the LPS-induced secretion of pro-inflammatory cytokines in vitro and in vivo [23,24,25]. The beneficial mechanisms of the cytoprotective effects of HO-1 are attributed essentially to its enzymatic action in pro-oxidant heme catabolism, and also to the heme catabolites [26,27]. However, many studies have suggested that the cytoprotective effects of HO-1 might occur via mechanisms independent from its enzymatic activity [28]. A recent study analyzed the subcellular localization of HO-1 and its role in oxidative stress and cell proliferation as a mediator of signaling function. This nuclear translocation of HO-1 was associated with the enhanced activation of oxidant-responsive transcription factors [29].

In this study, we investigated the effect of ELM-EMF exposure on HO-1 expression and its subcellular localization. By immunocytochemistry technique, we observed a significant increase in HO-1 nuclear staining in LPS-stimulated cells after 6 h of exposure to 50 Hz, 1 mT. By using western blot analysis, we confirmed that this induction was related to HO-1 nuclear translocation.

The upregulated HO-1 expression was linked to a fast activation of Nrf2 pathway, as shown by the phosphorylation and nuclear translocation of Nrf2 after 1 h of exposure, and to an initial oxidative stress shown by an increase in ROS level and a decreased GSH/GSSG ratio, as indicators of intracellular redox status. In agreement with the study of Rizzardini et al., depletion of cellular GSH-level, and consequent activation of the redox-sensitive transcription factors like Nrf2, strengthens the expression of HO-1 in ELF-EMF-exposed THP-1 cells [30]. Silent information regulator 1 (SIRT1) is a nicotinamide adenine dinucleotide-dependent deacetylase that plays an important role in inflammation and in the oxidative stress response. The crosstalk between SIRT1 and nuclear factor kappa B (NF-kB) signaling in immunity system regulation has been previously reported [31]. SIRT1 is recovered in the cytoplasm and nucleus and can inhibit inflammatory responses by deacetylating the p65 subunit of NF-κB [32]. Here, we observed that short exposure to ELF-EMF decreases the p65 phosphorylation level and increases the expression of SIRT1, which is significantly reverted after 24 h of exposure.

Our results are in agreement with previous data showing that the downregulation of SIRT1 expression reduces Nrf2 protein expression and that the overexpression of SIRT1 can deacetylate Nrf2, increasing its stability and endorsing the transport of Nrf2 to the nucleus [33].

The field intensity can affect enzymatic activities by simulating receptor binding [3,34] and modifying phosphorylation of intracellular proteins (e.g., transcriptional regulatory factors and enzyme regulators [35,36]). Thus, we evaluated the role of the MAPK and Akt pathways, involved on the modulation of phase II gene. Our results indicate that Erk and Akt pathways are activated by ELM-EMF exposure, playing an important role in the induction of HO-1.

The treatment with PD980559, a highly specific inhibitor that blocks ERK activity, and with Akt inhibitor LY294002, decreased Nrf2 nuclear fraction and downregulated HO-1 expression.

The involvement of PI3K/Akt in Nrf2 activation is well documented in cellular protection against oxidative stress-mediated diseases and disorders, with involvement of signaling pathways PI3K-Akt-GSK3ß axis that regulate the phosphorylation of Nrf2 and its nuclear storage [37]. In contrast, the role of ERK in ARE sequences activation is still controversial. Some authors have reported that LPS-induced HO-1 mRNA and protein expression via activation of the transcription factor Nrf2 was independent from the ERK [38], while others have shown that HO-1 induction was via the Erk pathway in PC-12 cells treated with the phytochemical carnosol or in rat hepatoma cells treated with heavy metal arsenite [39,40]. Also, the anti-neuroinflammatory activity of the herbal medicine astragaloside (ASI) exerted the activation of Nrf2/HO-1 via ERK signaling pathway as a novel mechanism in LPS-stimulated microglia cells [41]. One possible interpretation for these diverging observations on signal pathways activation may stem from different inducers and cell types. Thus, ELF-EMF exposure can be considered as a weak or mild cellular stressor that activates regulatory mechanisms to reduce pro-inflammatory mediators [4,42]. The formation of ROS, which act as redox signaling messengers, is necessary for the functioning of cells, but if ROS are produced in excess, they can be harmful. Thus, cells have developed an antioxidant mechanism that includes enzymes and non-enzymatic antioxidants. Our results showed that exposure to ELF-EMF induced a production of free radicals at 1 h in order to promote cellular response to stress stimuli, and at 6 h, ROS formation was balanced by an efficient antioxidant system, with recovery of GSH levels. Glutathione is the main metabolite involved in determining the cellular redox state. It is realistic to assert that the relative sensitivity to oxidative stress and thus the reducing power is related to cell types. Based on our results, we assume that short exposure to ELF-EMF quickly restores equilibrium between free radicals and antioxidants by cellular homeostatic mechanisms and may modulate the cascade of inflammatory progression. Our results reinforce the evidence that ELF-EMFs may exert effects on immune response, contributing to the activation of macrophages, and changing immunological reactions, such as reactive oxygen species-formation, phagocytic activity, and the production of cytokines.

Since HO-1 induction in macrophages has been shown to functionally switch these cells from M1 phenotype, producing pro-inflammatory cytokines and ROS, to the anti- inflammatory M2 phenotype, producing anti-inflammatory cytokines [43], and we have observed a reduction of ROS production in THP-1 exposed to ELF-EMF, we can speculate that a short exposure to 50 Hz, 1 mT ELF-EMF may help the resolution of inflammation modulating the Nrf2 antioxidant pathway and HO-1 expression, which facilitates macrophage polarization toward M2 anti-inflammatory phenotype.

Thus, it is possible to hypothesize the efficacy of ELF-EMF in alleviating the symptoms and the progression of diseases supported by inflammation. The results reported here could suggest a promising use of ELF-EMFs as a new therapeutic approach, even though further studies on the molecular mechanisms underlying the action of the ELF-EMFs are needed.

## 4. Materials and Methods

### 4.1. In Vitro Exposure System

Exposures were performed with an ELF-EMF in vitro exposure system that was comprised of two identical exposure chambers—for active and sham exposures. A sinusoidal 50-Hz ELF-EMF at a flux density of 1 mT rms, and with sham exposures of ~7 mT, produced by an electromagnetic generator (Agilent Technologies model 33220A, Santa Clara, CA, USA, with stability higher than 1% both in the frequency and in the amplitude) was custom assembled. A current flow of 1.20 A (Ieff) passed through a 160-turn solenoid coil (22 cm length, 6 cm radius, copper wire diameter of 1.25 × 10^−5^ cm). The generator, which was connected to a power amplifier (NAD electronics Ltd., model 216, London, UK), and an oscilloscope (ISO-TECH model ISR658, Vicenza, Italy) dedicated to monitoring output signals from a Gaussmeter (MG-3D, Walker Scientific Inc., Worcester, MA, USA) were outside the incubator. The achieved MF flux density (1 mT rms) was continuously monitored using a Hall-effect probe connected to the Gaussmeter.

Identical currents were present in the sham chamber, but with the dual-winded coils turned in the opposite direction to generate the same thermal load (~1 W at 1 mT) without the generation of any ELF-EMF.

Preliminarily, to indicate the warning zones where there is not an optimal spread of ELF-EMF and to suggest areas of propagation where the cell culture is uniformly covered by the generated ELF-EMF, we simulated the propagation of ELF-EMF in a solenoid with the same features of our system. The simulation evidenced that the field uniformity is 98% near the center of the solenoid, where the field lines are parallel to its length. Thus, cell cultures were placed within this region of the solenoid.

Solenoids for active and sham exposures were located in plastic chambers magnetically shielded and installed in two different commercial CO_2_ incubators (HeraCell 240i, Thermo-Fisher Scientific, Waltham MA, USA) for the same times and conditions. The exposure system was computer controlled and analyzed by Software SW-U801-WIN, for automated and continuous generation and monitoring of coil currents, and temperatures were recorded by a two-channel thermometer (TM-925, Lutron, Coopersburg, PA, USA). No significant temperature changes were observed to be associated with the application of the ELF-EMF field (ΔT = 0.1 °C).

### 4.2. Cell Line Cultures and Exposure Conditions

The human monocytic leukemia cell line THP-1 was purchased from the American Type Culture Collection (Rockville, MD, USA) and maintained in Roswell Park Memorial Institute (RPMI) 1640 (Sigma) containing 10% heat-inactivated fetal bovine serum (Sigma), 2 mM l-glutamine, and 10 mM HEPES, at 37 °C in a humidified atmosphere of 5% CO_2_ and incubated with 10 μg/mL *Escherichia coli* LPS (Sigma). Cell cultures were located in the central part of the solenoid, which was characterized by the highest field homogeneity (98%). Simultaneously, another set of experiments (sham-exposed) was performed by placing THP-1 cells within the central region of an identical electrically disconnected solenoid. Cell viability was not influenced by ELF-EMF exposure, as checked by trypan blue day exclusion. Each experimental condition reported was performed in triplicate and reproduced at least three times

### 4.3. Western Blot Analysis

The nuclear, cytoplasmatic, and total protein fraction, from THP-1 cells, were separately quantified by Bradford Assay and analyzed by western blot, using the following antibodies:HO-1 (sc-136960), lamin B (sc-374015), SIRT-1 (sc-74465), p-p65 (Ser 536, sc-33020), p65 (sc-109), Akt (G-5, sc-55523), p-Akt (Thr 308, sc-135650), p-ERK (Thr202 sc-101760), ERK (sc-271269), and β-actin (A5441; Sigma-Aldrich). Signals were detected by Super Signal Ultra chemiluminescence reagents (Pierce Biotechnology, Rockford, IL, USA). The blot images were analyzed with a gel analysis software package (Gel Doc 1000; Bio-Rad).

### 4.4. RNA Extraction, RT and Real-Time PCR

Total RNA was extracted from THP-1 using QIAzol reagent (Qiagen, Hilden, Germany) according to the manufacturer’s protocol. The RNA concentration was determined by measuring the samples’ absorbance at 260 nm by NanoDrop 2000 UV-Vis Spectrophotometer (Thermo Scientific, Waltham, MA, USA), and its purity was assessed by the absorbance ratio 260/280 nm and 260/230 nm. For each sample, 1 µg of RNA was reverse transcribed into complementary DNA (cDNA) using QuantiTect Reverse Transcription Kit (Qiagen, Hilden, Germany). Subsequently, Real-Time PCR was performed using the GoTaq^®^ qPCR Master Mix (Promega, Madison, WI, USA) to evaluate the gene expression of HO-1 and RPS18, used as reference gene. All PCR reactions were performed in triplicate using the Mastercycler ep (Eppendorf, Hamburg, Germany) with the following conditions: initially, 2 min of incubation at 95 °C, followed by 40 cycles consisting of 30 s at 95 °C, then 60 °C for 1 min and 30 s at 68 °C. The gene expression analysis was carried out according to ΔΔCt method [44].

### 4.5. Immunofluorescence Staining and Confocal Laser Scanning Microscope Analysis

ELF-EMF-exposed and sham-exposed cells were collected and fixed with 4% paraformaldehyde for 30 min at room temperature and permeabilized with 0.1% Triton-X100 in Phosphate-buffered saline (PBS) for 5 min. For immunostaining analysis, the cell suspensions were cytospinned to glass slides at 1000 rpm × 5 min (Shandon cytospin™^3^ Cytocentrifuge, Thermo Scientific, Milan, Italy). The blocking step was performed by 5% of milk in PBS for 1 h at rate temperature, and subsequently, the samples were incubated with 1:200 rabbit primary monoclonal antibody, anti-heme oxygenase-1 (Santa Cruz Biotechnology, Inc., Dallas, TX, USA) followed by anti-rabbit Alexa Fluor 568 (Life Technologies, Monza, Carlsbad, CA, USA) and with TO-PRO staining to highlight the nuclei. The images were captured by CLSM (Zeiss LSM510 META, Zeiss, Jena, Germany), equipped with a Plan Neofluar oil-immersion objective (63 × /1.4 NA) [45]. The co-localization analysis on the nuclei was performed offline on images acquired at a resolution of 1024 × 1024 pixels at 12 bit (4096 grey values) using Zen2010 software (Zeiss) and expressed by Pearson’s correlation coefficient calculation.

### 4.6. ROS Quantification

The amount of intracellular ROS was quantified by 2′,7′-dichlorodihydrofluorescein diacetate (H_2_DCF-DA) probe. The cells were seeded at 2.5 × 10^5^ cells/mL and, at the end of each ELF-EMF-exposure, cells were incubated for 30 min at 37 °C with H_2_DCF-DA (5 µM). Fluorescence intensity of H_2_DCF-DA was measured by a fluorescence spectrophotometer using excitation/emission 485/530 nm. Time-zero fluorescence was measured to normalize results versus a live cell number. Data are expressed as the fold increases in ELF-EMF-exposed samples with respect to the sham-exposed control.

### 4.7. Glutathione Assay

GSH and GSSG levels were measured by enzymatic assay as described previously [46]. Briefly, THP-1 cells were suspended in extraction buffer (ascorbic acid (10 mM), HCl (20 mM), trichloroacetic acid (5%), and diethylene triamine pentaacetic acid (5 mM) (Sigma-Aldrich, St. Louis, MO, USA). The extracts were centrifuged (12,000× *g*) for 5 min at 4 °C, and the supernatants were used to determine GSH and GSSG levels. To measure GSSG levels, the GSH present in the sample was obtained by adding 2 mL of 2-vinylpiridine and 6 mL of triethanolamine to a 100-mL aliquot of supernatant. The fluorescence was measured using a Plate Reader (Bio-Rad, Hercules, CA, USA) at an excitation wavelength of 355 nm and an emission wavelength of 430 nm. The amounts of GSH and GSSG were determined using a standard curve.

### 4.8. Glutathione Peroxidase (GPX) and Glutathione Reductase (GR) Activity

GPX activity was measured spectrophotometrically with H_2_O_2_ (0.25 mM) as substrate, recording the decrease of absorbance for NADPH oxidation at 340 nm, according to a previously described method [46]. One unit of enzyme activity was defined as 1 µmol of NADPH oxidized/min at 25 °C.

The GR activity was determined spectrophotometrically at 340 nm and 25 °C, as described previously [46]. One unit of enzyme activity was defined as 1 mmol of NADPH oxidized/min at 25 °C.

### 4.9. Statistical Analysis

All results were expressed as mean ± standard deviation. For repeated measures, ANOVA was performed to compare means between groups. The ‘fold change’ of gene expression levels was calculated with the 2^−ΔΔCt^ method. The hypothesis that the fold change between exposed and not exposed was equal to 1 was tested with the Student’s *t*-test for unpaired data.

The data of immunofluorescence staining and confocal laser scanning microscopy were analyzed using Prism 5.0 (GraphPad Software, La Jolla, CA, USA). One-way repeated-measurement analysis of variance (ANOVA), followed by the post hoc “Bonferroni’s Multiple Comparison Test” (colocalization test), was applied. In all statistical tests, the threshold of statistical significance was assumed equal to *p* ≤ 0.05 (*). Analyses were performed by SPSS Inc. (Chicago, IL, USA) statistical software package (Version 23.0).

## Supplementary Materials

Supplementary materials can be found at https://www.mdpi.com/1422-0067/21/19/7284/s1.

## Abbreviation

reactive oxygen species	(ROS)
heme oxygenase-1	(HO-1)
electromagnetic fields	(EMFs)
extremely low frequencies	(ELFs)
matrix metalloprotease	(MMP)9
mammalian target of rapamycin	(mTOR)
phosphoinositide 3-kinases/protein kinase B	(PI3K/Akt)
extracellular signal-regulated kinases	(ERK)
nitric oxide synthetase	(NOS)
catalase	(CAT)
cytochrome	(CYP) 450
lipopolysaccharides	(LPS)
nuclear factor erythroid 2-related factor 2	(Nrf2)
NAD-dependent deacetylase sirtuin-1	(SIRT1)
intracellular reduced glutathione	(GSH)
oxidized glutathione	(GSSG)
glutathione peroxidase	(GPx)
glutathione reductase	(GR)
