# Supplementary material for: Short ELF-EMF Exposure Targets SIRT1/Nrf2/HO-1 Signaling in THP-1 Cells
Source: Int J Mol Sci. 2020 Oct 2;21(19):7284. doi: 10.3390/ijms21197284 (PMC7582394; doi:10.3390/ijms21197284)
Supplement: Supplementary file 1 [file ijms-21-07284-s001.zip › ijms-925030-supplementary.pptx]

## Slide 1
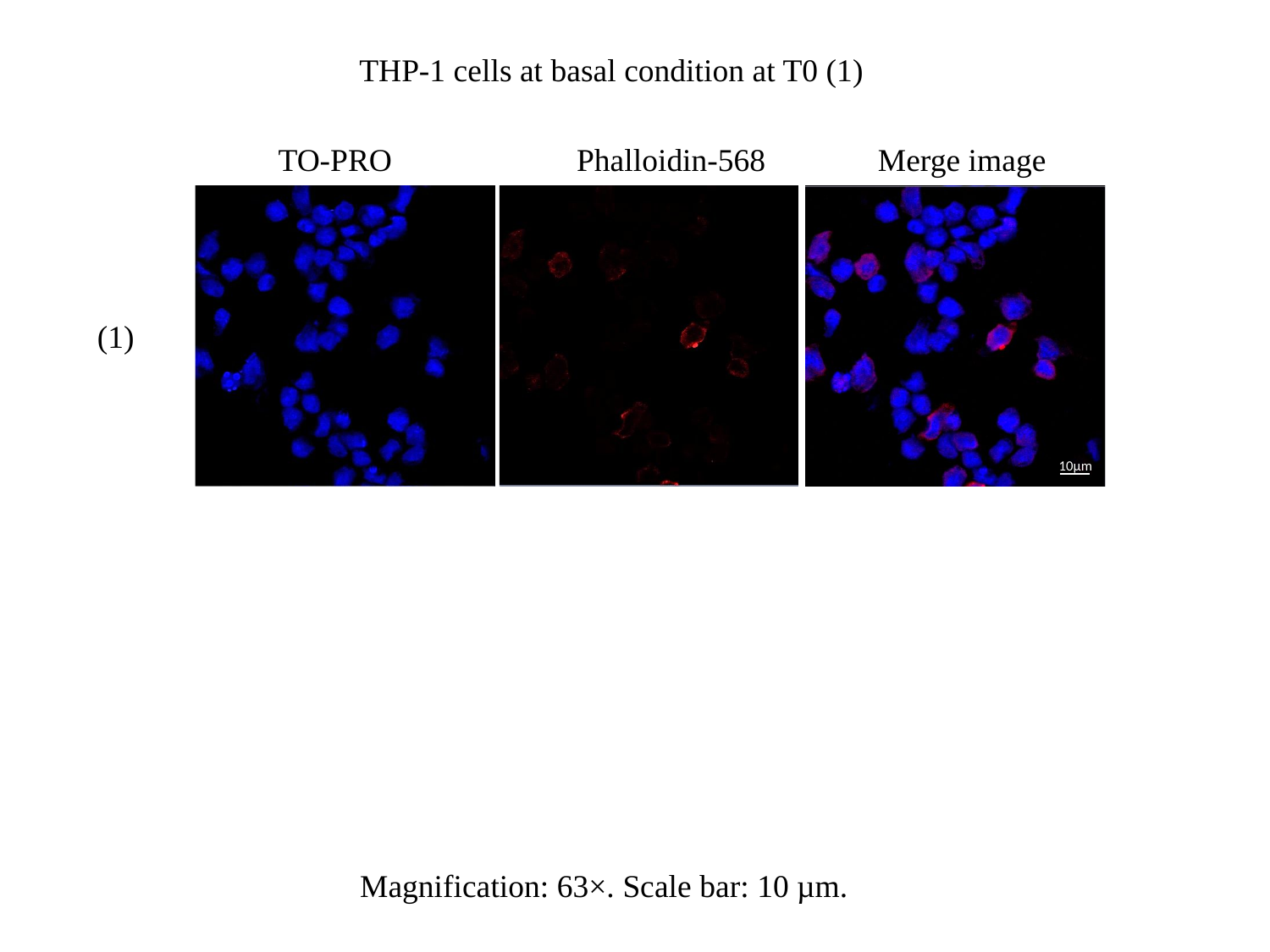

THP-1 cells at basal condition at T0 (1)
 TO-PRO Phalloidin-568 Merge image
10µm
(1)
Magnification: 63×. Scale bar: 10 µm.

## Slide 2
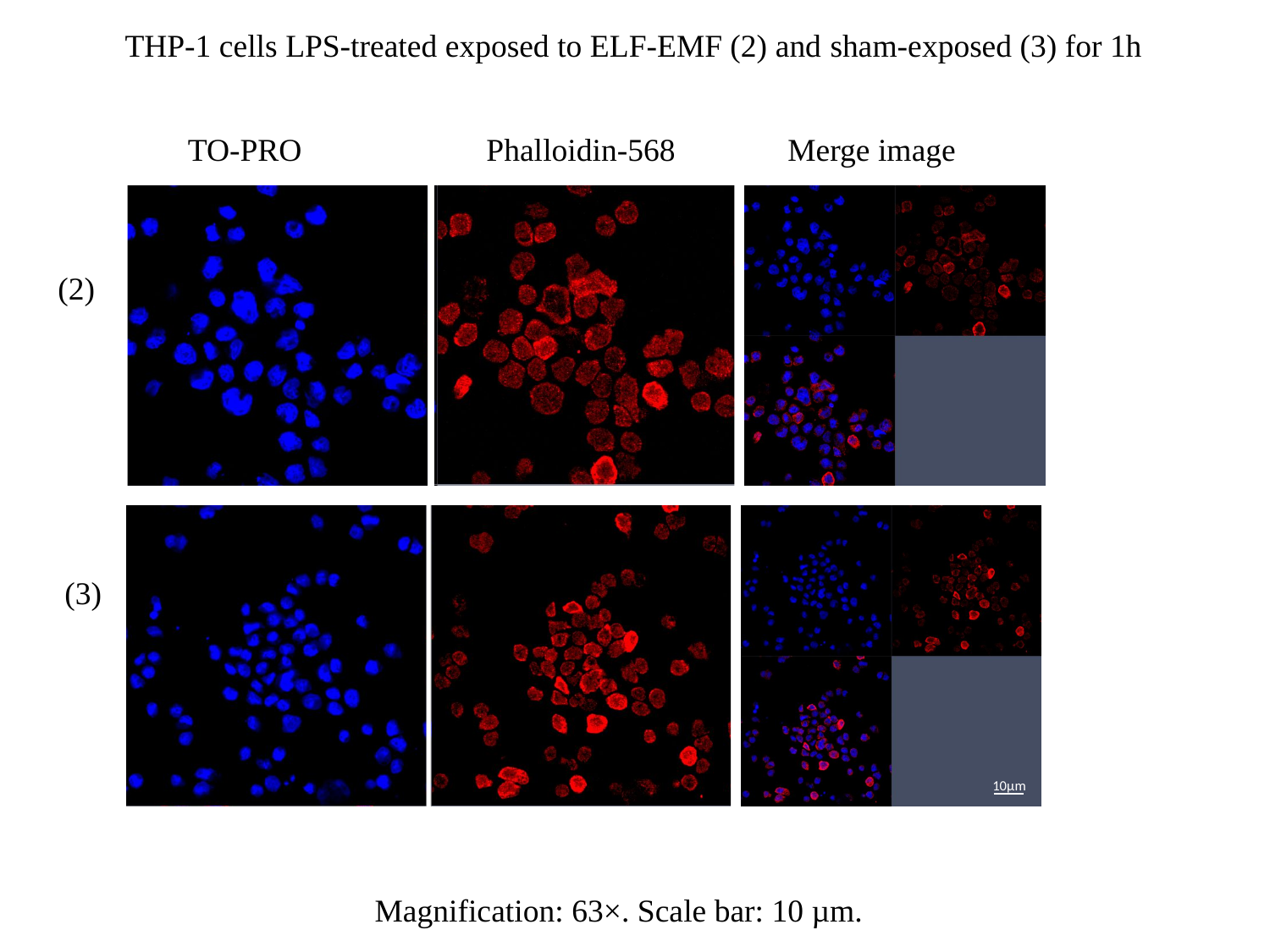

THP-1 cells LPS-treated exposed to ELF-EMF (2) and sham-exposed (3) for 1h
 TO-PRO Phalloidin-568 Merge image
(2)
(3)
10µm
Magnification: 63×. Scale bar: 10 µm.

## Slide 3
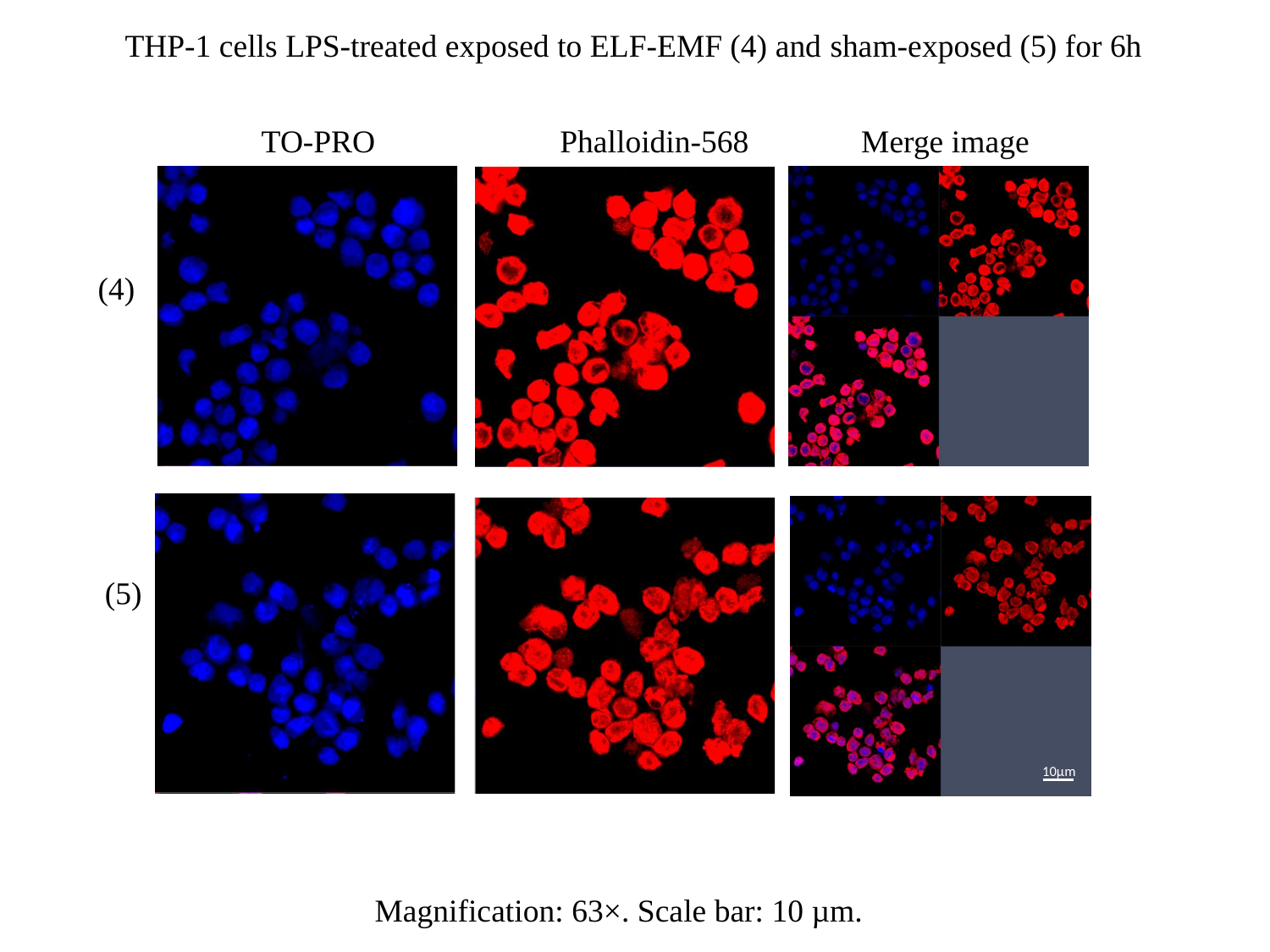

THP-1 cells LPS-treated exposed to ELF-EMF (4) and sham-exposed (5) for 6h
 TO-PRO Phalloidin-568 Merge image
(4)
(5)
10µm
Magnification: 63×. Scale bar: 10 µm.

## Slide 4
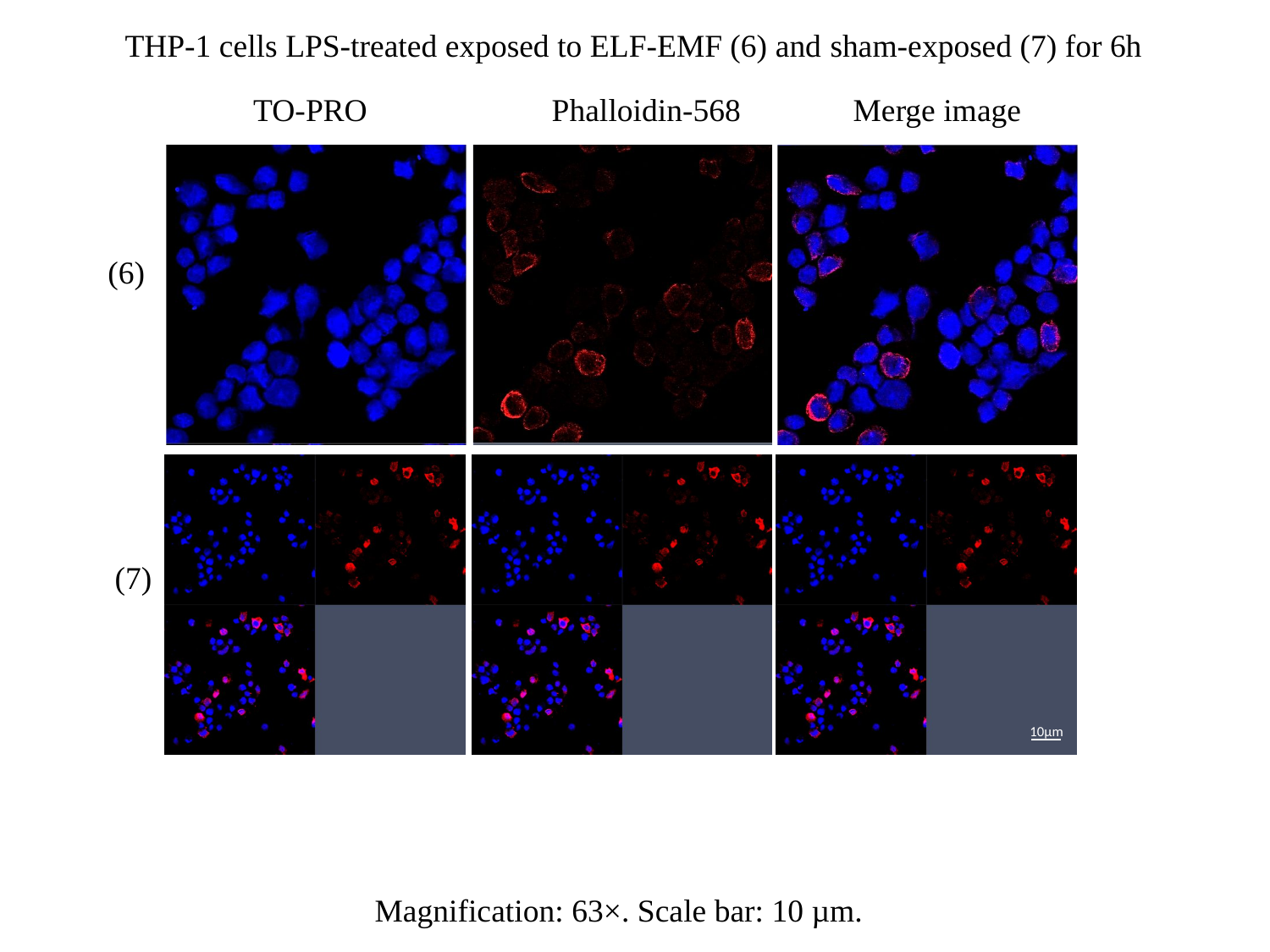

THP-1 cells LPS-treated exposed to ELF-EMF (6) and sham-exposed (7) for 6h
 TO-PRO Phalloidin-568 Merge image
(6)
(7)
10µm
Magnification: 63×. Scale bar: 10 µm.
